# Cholera—Dr. Bird and His Sulphur Pills

**Published:** 1849-08

**Authors:** 


### CHOLERA—DR. BIRD AND HIS SULPHUR PILLS.

It appears that Dr. Bird is about to distinguish himself still further, in his much vaunted specific—sulphur.

His pills, it seems, have shown themselves a trifle too potent, having produced in a young lady, at Detroit, in addition to the arrest of the diarrhea, unmistakable narcotism—an effect hitherto, we believe, never attributed to sulphur.

The Detroit Free Press, for June 16, contains the following curious statement, by Dr. Z. Pitcher, respecting the Chicago Remedy:

"I was called," says Dr. P., "to see a young lady, quite indisposed, whose symptoms were so analogous to those produced by a full dose of opium or morphine, that the inquiry was at once made of her friends whether she had taken a narcotic in any form, within the last few hours.

Both by them and herself I was assured, that the only medicine she had taken in several days, was one of Dr. Bird's Chicago pills, the evening before, to arrest a diarrhea, which it had done very effectually.

"I am so strongly impressed with the idea that the symptoms of narcosis were occasioned by the pill, that I have obtained two others from the same source."

These last two, unfortunately for Dr. Bird and the sulphur, were submitted to A. R. Terry, M. D., who, upon chemical analysis, says he discovered *a notable portion of morphia,* or one of its salts! Dr. Terry, not satisfied with this, went still further, and "took," says he, "the pains to trace these pills, and have the strongest reasons to suppose—nay, I am sure—that they came from Dr. Bird, of Chicago, himself."

